# Effects of schistosomiasis on susceptibility to HIV-1 infection and HIV-1 viral load at HIV-1 seroconversion: A nested case-control study

**DOI:** 10.1371/journal.pntd.0005968

**Published:** 2017-09-25

**Authors:** Jennifer A. Downs, Kathryn M. Dupnik, Govert J. van Dam, Mark Urassa, Peter Lutonja, Dieuwke Kornelis, Claudia J. de Dood, Pytsje Hoekstra, Chifundo Kanjala, Raphael Isingo, Robert N. Peck, Myung Hee Lee, Paul L. A. M. Corstjens, Jim Todd, John M. Changalucha, Warren D. Johnson, Daniel W. Fitzgerald

**Affiliations:** 1 Center for Global Health, Department of Medicine, Weill Cornell Medicine, New York, New York, United States of America; 2 Department of Medicine, Bugando Medical Centre, Mwanza, Tanzania; 3 Department of Parasitology, Leiden University Medical Center, Leiden, the Netherlands; 4 National Institute for Medical Research, Mwanza, Tanzania; 5 Department of Molecular Cell Biology, Leiden University Medical Center, Leiden, the Netherlands; 6 Department of Applied Biostatistics, London School of Hygiene and Tropical Medicine, London, United Kingdom; George Washington University, UNITED STATES

## Abstract

**Background:**

Schistosomiasis affects 218 million people worldwide, with most infections in Africa. Prevalence studies suggest that people with chronic schistosomiasis may have higher risk of HIV-1 acquisition and impaired ability to control HIV-1 replication once infected. We hypothesized that: (1) pre-existing schistosome infection may increase the odds of HIV-1 acquisition and that the effects may differ between men and women, and (2) individuals with active schistosome infection at the time of HIV-1 acquisition may have impaired immune control of HIV-1, resulting in higher HIV-1 viral loads at HIV-1 seroconversion.

**Methodology/Principal findings:**

We conducted a nested case-control study within a large population-based survey of HIV-1 transmission in Tanzania. A population of adults from seven villages was tested for HIV in 2007, 2010, and 2013 and dried blood spots were archived for future studies with participants’ consent. Approximately 40% of this population has *Schistosoma mansoni* infection, and 2% has *S*. *haematobium*. We tested for schistosome antigens in the pre- and post-HIV-1-seroconversion blood spots of people who acquired HIV-1. We also tested blood spots of matched controls who did not acquire HIV-1 and calculated the odds that a person with schistosomiasis would become HIV-1-infected compared to these matched controls. Analysis was stratified by gender. We compared 73 HIV-1 seroconverters with 265 controls. Women with schistosome infections had a higher odds of HIV-1 acquisition than those without (adjusted OR = 2.8 [1.2–6.6], p = 0.019). Schistosome-infected men did not have an increased odds of HIV-1 acquisition (adjusted OR = 0.7 [0.3–1.8], p = 0.42). We additionally compared HIV-1 RNA levels in the post-seroconversion blood spots in HIV-1 seroconverters with schistosomiasis versus those without who became HIV-infected in 2010, before antiretroviral therapy was widely available in the region. The median whole blood HIV-1 RNA level in the 15 HIV-1 seroconverters with schistosome infection was significantly higher than in the 22 without schistosomiasis: 4.4 [3.9–4.6] log_10_ copies/mL versus 3.7 [3.2–4.3], p = 0.017.

**Conclusions/Significance:**

We confirm, in an area with endemic *S*. *mansoni*, that pre-existing schistosome infection increases odds of HIV-1 acquisition in women and raises HIV-1 viral load at the time of HIV-1 seroconversion. This is the first study to demonstrate the effect of schistosome infection on HIV-1 susceptibility and viral control, and to differentiate effects by gender. Validation studies will be needed at additional sites.

## Author summary

Schistosomiasis is a neglected tropical parasitic worm infection that affects 218 million people worldwide, the majority of whom live in Africa. Studies have shown that women with schistosomiasis tend to have higher rates of HIV infection, but until now no study has followed people over time to determine whether schistosomiasis increases a person’s risk of becoming HIV-infected. In this ongoing community study in rural Tanzania, we identified 73 individuals who became HIV-infected during follow-up, and 265 control individuals who were similar in age, sex, and location but who remained HIV-uninfected during follow-up. We tested these individuals’ stored blood samples to determine whether they had schistosome infection before they became HIV-infected. We found that women who had schistosome infections had a 2.8-fold increased risk of becoming HIV-infected compared to women without schistosome infections. We found no evidence of increased HIV acquisition in men with schistosome infections. We also found that the concentration of HIV in the blood shortly after people became HIV-infected was higher in those who had schistosome infections than in those who did not have schistosome infections. Our study suggests that schistosomiasis may play a major role in HIV transmission and disease progression in African countries.

## Introduction

Schistosomiasis is a parasitic worm infection acquired by contact with contaminated fresh water. Over 90% of the world’s 218 million schistosome infections occur in Africa [1]. Schistosome worms live in the human host’s pelvic and gastrointestinal venules and daily lay hundreds of eggs that migrate to the urogenital and gastrointestinal mucosa. These eggs cause mucosal inflammation and physical breaches in the mucosa. These schistosome-induced changes are postulated to alter host susceptibility and immune control of HIV-1 infection [2,3].

Three cross-sectional studies in Tanzania and Zimbabwe have demonstrated increased prevalence of HIV infection in women with *Schistosoma haematobium* [4,5] or *S*. *mansoni* [6] infection. In our studies in Mwanza, Tanzania, women with *S*. *haematobium* infection were four times more likely to be HIV-1-infected than women without schistosomiasis, and women with *S*. *mansoni* were six times more likely [4,6]. These epidemiologic studies were conducted in women because the eggs of *S*. *haematobium* and *S*. *mansoni* in the female genital tract cause inflammation and ulceration and have been hypothesized to facilitate HIV-1 viral entry following sexual exposure [7–9]. Genital lesions are less common in men [10,11].

Macaque studies suggest that *S*. *mansoni* infection may increase susceptibility to HIV infection and increase HIV-1 RNA viral load levels in those who become HIV-infected. Macaques with and without pre-existing *S*. *mansoni* infection were rectally inoculated with progressively-increasing doses of simian HIV (sHIV). Macaques with *S*. *mansoni* infection developed systemic sHIV infection at a dose 17 times lower than macaques without schistosomiasis [12]. The investigators observed no difference in sHIV susceptibility when these experiments were repeated using intravenous inoculation of sHIV rather than inoculation via the rectal mucosa [13]. This implies that schistosome infection may alter mucosal integrity, thereby increasing susceptibility to trans-mucosal HIV infection.

In addition, two studies have found that macaques with pre-existing *S*. *mansoni* infection developed higher sHIV viral loads for the first 10 to 28 weeks post-sHIV inoculation than macaques without schistosomiasis [12,14]. Again, this effect on viral load was only seen when macaques were infected rectally and not when they were infected intravenously [13]. In humans, HIV-1 viremia peaks 6–18 days after acute infection [15]. As host HIV-1 specific immune responses develop, viremia is reduced to a point of equilibrium between host and virus, leading to a stable viral load set-point within 6 weeks [15]. Set-points vary by several orders of magnitude between individuals and are influenced by host genetics, host immune factors, and viral genetics [16]. Elevated set-points lead to increased HIV-1 transmission and more rapid progression to AIDS and death [17,18].

Our goal was to determine whether schistosome infection affects susceptibility to HIV-1 acquisition and HIV-1 viral load at the time of HIV-1 seroconversion. We therefore conducted a nested case-control study within a large ongoing population-based survey of HIV-1 transmission in northwest Tanzania. We analyzed dried blood spots that had been stored prospectively to test our hypotheses that: (1) pre-existing schistosome infection may increase the odds of HIV-1 acquisition and that the effects may differ between men and women, and (2) individuals with active schistosome infection at the time of HIV-1 acquisition may have impaired immune control of HIV-1, resulting in higher HIV-1 viral loads at the time of HIV-1 seroconversion.

## Methods

### Ethics statement

This project was approved by Bugando Medical Centre (Mwanza, Tanzania, BREC/001/04/2011), the National Ethical Review Board (Dar es Salaam, Tanzania, NIMR/HQ/R8.a/Vol.IX/1313), and Weill Cornell Medicine (New York, USA, 1108011883). Written informed consent was obtained from study participants, and consent from parents of those aged 15 to 17 years with assent of the minor was obtained. Study participants also provided consent for future testing of dried blood spot samples in accordance with approved procedures of the Kisesa cohort study [19]. Dried blood spot samples were stored anonymously and unlinked from personal identifiers.

### Study area

Since 1994, the Kisesa observational HIV-1 cohort study has serially surveyed and HIV-tested community members living in the Kisesa Ward in the Magu District of northwest Tanzania [20]. The Kisesa study area includes seven villages located near Lake Victoria. Consenting adolescents and adults aged 15 and above are tested for HIV infection every 3 years. They receive free voluntary HIV counseling and testing with same-day results and participate in structured interviews. Dried blood spots are also archived for future studies with study participants’ consent. This ongoing study is operated by the TAZAMA Project under the Tanzanian National Institute for Medical Research in Mwanza.

The current study utilized data from archived dried blood spots collected from participants in the Kisesa cohort during sero-surveys in 2007, 2010, and 2013. Sociodemographic data from the same time points were also available. In this area of Tanzania, ~40% of adults have *S*. *mansoni* infection and 2% have *S*. *haematobium* infection [6,21,22]. In our other studies, less than one-fourth of adults in this region have reported receiving praziquantel treatment in the past five years [6,22].

### Study design

#### Study of HIV-1 susceptibility

To test our hypothesis that individuals with schistosome infection were at higher risk of HIV-1 acquisition, we conducted a case-control study nested within the Kisesa cohort. We identified HIV-1 seroconverters from two successive surveys conducted by the cohort study: those whose dried blood spots tested negative for HIV in 2007 and positive for HIV-1 in 2010, and those whose dried blood spots tested negative for HIV in 2010 and positive for HIV-1 in 2013. HIV-1 seroconverters were selected randomly from among all HIV-1 seroconverters identified during successive surveys. For each HIV-1 seroconverter, we sought four controls who also had dried blood spots available and were HIV-negative at the same two time points, and matched them to cases by gender, village, and age (within 5 years if age < 35, and within 10 years if ≥ 35). We compared proportions of schistosome antigen CAA-positivity between cases and controls. Results were stratified by gender.

#### Study of HIV-1 viral loads at time of HIV-1 seroconversion

To test our hypothesis that individuals with schistosome infection at the time of HIV-1 acquisition would have higher viral loads at the time of HIV-1 seroconversion, we identified HIV-1 seroconverters who were HIV-negative in 2007 and HIV-1-positive in 2010. We restricted our analysis to this time point because in 2012 antiretroviral therapy became widely available in the region [23,24]. Because seroconverters had become HIV-1-infected during the three-year period since their prior negative HIV test and because the viral load set-point is generally stable from 6 weeks until at least 24 months post-infection and typically longer [25,26], we assumed that the HIV-1 RNA level measured would be reflective of the HIV-1 RNA viral load set-point in most HIV-1 seroconverters. We quantified schistosome circulating anodic antigen (CAA) in the dried blood spots collected before and after HIV-1 seroconversion, and measured HIV-1 RNA levels in the dried blood spots collected at the time the HIV-1 seroconversion was identified. We compared HIV-1 viral loads between people with and without schistosome infection at the time of HIV-1 acquisition.

#### Study definitions

We defined an individual as schistosome-infected at the time of HIV-1 seroconversion if the dried blood spots collected both before and after HIV-1 seroconversion tested positive for schistosome CAA. We defined the viral load at the time of HIV-1 seroconversion as the number of copies/mL of HIV-1 RNA in whole blood from the first dried blood spot at which a participant was identified as HIV-1-sero-positive.

### Laboratory testing

#### Specimens

Capillary blood was collected by finger prick directly onto the five sample spots of a Whatman Protein Saver 903 card (GE Healthcare Bio-Sciences, Pennsylvania). Each sample spot contains ~80 μL of blood. Blood spot cards were dried out of direct sunlight and then sealed in a gas-impermeable zip-bag with desiccant and humidity indicator. Dried blood spots were tested for HIV at the National Institute for Medical Research laboratory in Mwanza using the 4^th^-generation Vironostika Uni-Form Antigen/Antibody test (Organon Teknika, the Netherlands) with Enzygnost Anti-HIV1/2 Plus (Dade Behring, Germany) for confirmation of positives. All laboratory analysis was performed by technicians who were blinded to other results.

#### CAA quantification

Circulating anodic antigen (CAA) is a glycosaminoglycan-like carbohydrate regurgitated by adult schistosome worms into the host bloodstream [27,28]. CAA antigen levels are directly proportional to the worm burden in the host [29,30]. CAA is a stable molecule, detectable in serum and dried blood spots, that does not differentiate between schistosome species [21,29]. We quantified CAA in dried blood spots as previously described [21]. Briefly, a 216 mm^2^ area of dried blood spot was cut from the card, eluted in phosphate-buffered saline overnight, and concentrated using a 10 kDa concentration device (Amicon Ultra–0.5mL Centrifugal Filters, Millipore). Immunochromatography, scanning of lateral flow test strips, and calculation of CAA concentrations were performed at Leiden University Medical Center with a lower limit of quantitation of the CAA assay of 2 pg/mL (Leiden, the Netherlands).

#### Quantification of whole blood HIV-1 RNA

The Abbott system was used to quantify whole blood HIV-1 RNA copies per mL in dried blood spots, as previously described [31–33]. This system requires one full dried blood spot (133 mm^2^, containing 80 μL blood), which was placed application side inwards into a 2 mL microtube. 1.1 mL of Bulk Lysis Buffer GPR (Abbott, Illinois) was added to the dried blood spot and vortexed. After room-temperature incubation for one hour with intermittent vortexing, the lysate (800 μL) was placed into a new tube. RNA was extracted from 600 μL of lysate using the *m*Sample Preparation System and quantified by *m*2000 Real-Time HIV-1 assay (Abbott, Illinois), per the manufacturer’s instructions. To ensure specimen quality, all samples were run with an internal positive control and each PCR run included negative, low-positive, and high-positive *m*2000 controls, per the manufacturer's recommendations. An additional extraction positive control for all runs was a dried blood spot replicate from the same HIV-1-positive sample.

The Abbott *m*2000 machine reports viral load results as copy numbers per mL of extracted lysate. Whole blood viral load was calculated by multiplying the value obtained from the input lysate by 10 (to account for the 80:800 dilution of whole blood in lysis buffer) [33]. The lower limit for quantification by quantitative PCR is 400 copies/mL. Detectable viral load values below 400 copies/mL were recorded as 399 copies/mL, and undetectable viral loads were recorded as 1 copy/mL [31,32].

### Statistical considerations

Data were analyzed using Stata/IC 14 (StataCorp, Texas, USA). Binary variables were described as proportions and continuous variables were described as medians [interquartile ranges]. Proportions were compared by Chi-square or Fisher’s exact test and medians by Wilcoxon rank-sum test.

#### Analysis of HIV-1 susceptibility

We used univariable conditional logistic regression to calculate odds ratios for subsequent HIV-1 acquisition among individuals with versus without schistosome infection, while accounting for matching. Variables in the conditional logistic regression models were chosen by a backward selection procedure that began with all variables and used an elimination criteria of p>0.1. We pre-specified that we would analyze the data stratified by gender due to postulated gender-specific mechanisms of HIV-1 susceptibility [2].

#### Analysis of viral loads at HIV-1 seroconversion

To assess the relationship between the logarithm of the HIV-1 RNA viral load at HIV-1 seroconversion and schistosomiasis, we used a multivariable tobit regression analysis with a lower limit of log_10_ (399) to account for left-censored data. To account for variance in the viral loads across villages, we used a random effects tobit regression. The results from the tobit analysis are interpreted as the increased log_10_ viral load in those with schistosomiasis, weighted by the proportion seen with a viral load of 400 copies/ml or more. We compared all models using the likelihood ratio test.

Due to the limited and irreplaceable nature of dried blood spots, we tested only the minimum number of samples required according to pre-specified power calculations at 5% significance. We calculated that 74 HIV-1 seroconverters and four matched controls would provide >80% power to detect an odds ratio of 3 for HIV-1 infection, stratified by gender. We calculated that viral load quantification in 37 HIV-1 seroconverters would provide 83% power to detect a difference of 0.5 log_10_ HIV-1 RNA copies/mL in those with versus without schistosome infection. There was an insufficient number of HIV-1 seroconverters in 2010 to power an analysis of viral loads stratified by gender.

## Results

### Study population and selection of dried blood spots

**Fig 1:**
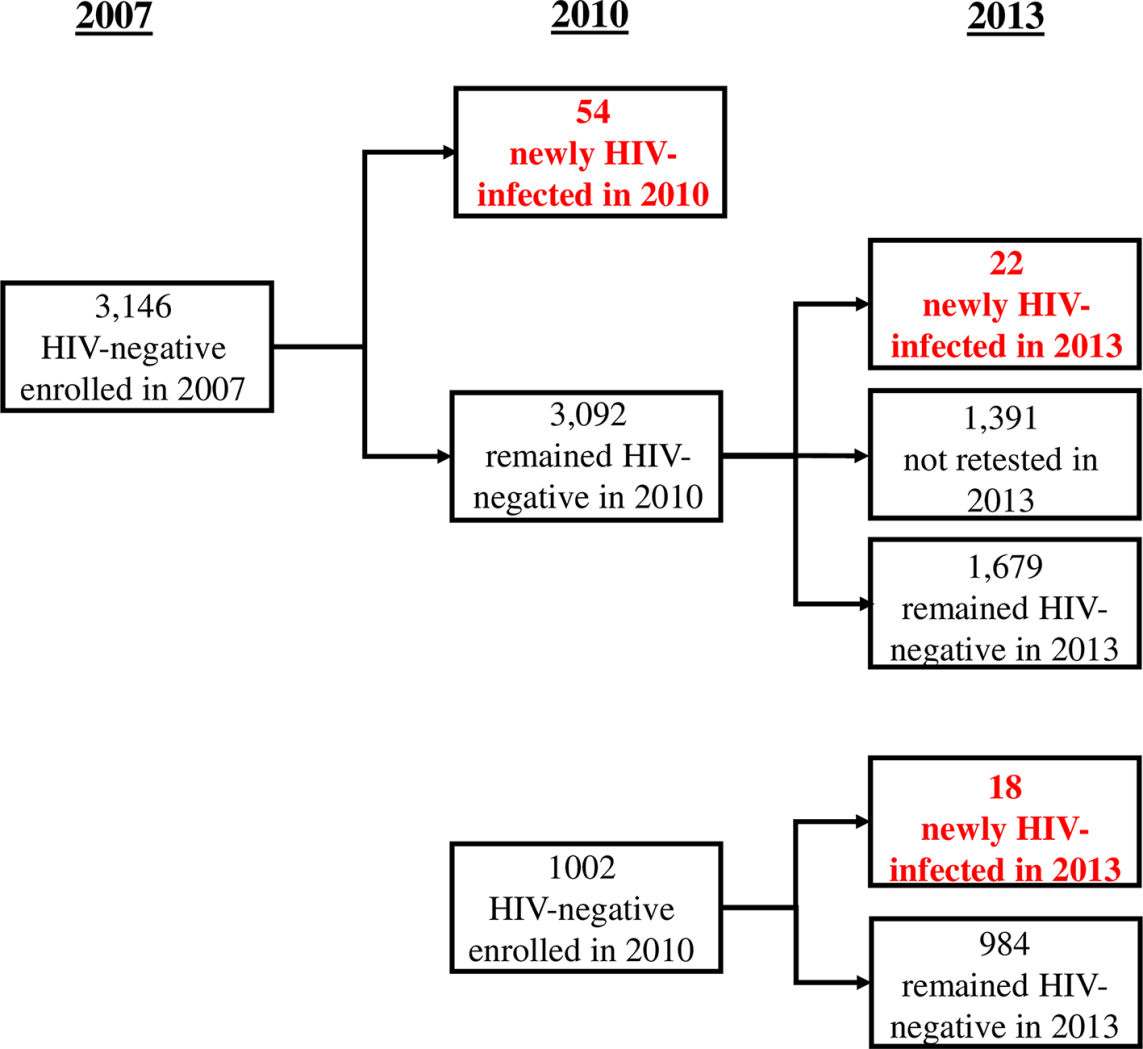
Flow diagram depicting identification of HIV-1 seroconverters and controls who remained HIV-uninfected. The numbers of people tested in 2007, 2010, and 2013 in the Kisesa HIV-1 observational cohort study are detailed above. Incident cases identified in 2010 and 2013 are indicated with red font. Dried blood spots from 73 of these HIV-1 seroconverters were utilized in the current studies. We also analyzed dried blood spots from 265 HIV-uninfected matched controls who were repeat tested but remained HIV-seronegative.

In 2010, 3,146 adults who were HIV-uninfected in 2007 were re-tested and 54 were found to have HIV-1-seroconverted during the three-year interval. In 2013, 2,701 adults who had been HIV-uninfected in 2010 were re-tested and 40 had newly HIV-1-seroconverted (**Fig 1**). We obtained dried blood spots for schistosome testing from 37 of the 2010 seroconverters and 37 of the 2013 seroconverters who were randomly selected from among all HIV seroconverters. One blood spot from a 2013 HIV-1 seroconverter was lost accidentally in the laboratory. Controls were selected to match the HIV-1 seroconverters at each time point.

### Study of HIV susceptibility

For the case-control study, we were able to identify 4 controls for 55 cases, 3 controls for 9 cases, and 2 controls for 9 cases, yielding a total of 73 HIV-1 seroconverters and 265 HIV-uninfected controls. Cases and controls had a median age of 35 [interquartile range, 25–43] years and 34 [interquartile range, 25–44] years, respectively. Women comprised 61% of cases (45/73) and controls (162/265). Cases were included from all seven of the villages in the TAZAMA project, with a range of 4 to 18 cases and their matched controls coming from each village. Characteristics of HIV-1-infected cases and HIV-negative controls, stratified by gender, are shown in **Table 1**.

**Table 1:** Demographic and behavioral characteristics of HIV-1-infected cases and HIV-uninfected controls, by gender.

Characteristic	HIV-1-infected women (n = 45)	HIV-uninfected women (n = 162)	p-value for women	HIV-1-infected men (n = 28)	HIV-uninfected men (n = 103)	p-value for men
Years of school attended	7 [7–7]	7 [7–7]	0.40	7 [7–7]	7 [6–7]	0.89
Marital status Never married or cohabited Monogamously married / cohabiting Polygamously married / cohabiting Widowed / separated / divorced	8 (18.6) 17 (37.8) 4 (9.3) 16 (37.2)	27 (17.3) 98 (62.8) 10 (6.4) 21 (13.5)	0.82 **0.010** 0.51 **0.001**	3 (10.7) 21 (75.0) 1 (3.6) 3 (10.7)	27 (26.7) 60 (59.4) 11 (10.9) 3 (3.0)	**0.08** 0.19 0.46 0.12
Occupation Farming Small business Student Other / none	34 (75.6) 6 (13.3) 2 (4.4) 3 (6.7)	125 (78.6) 9 (5.7) 14 (8.8) 14 (8.6)	0.84 0.10 0.53 1.0	24 (85.7) 0 1 (3.6) 3 (10.7)	85 (82.5) 1 (1.0) 12 (11.6) 5 (4.9)	0.78 1.0 0.30 0.37
Age in years at first sex	17 [15–18]	17 [15–19]	0.29	19 [15–21]	18 [17–21.5]	0.97
Number of lifetime sexual partners	3 [2–4]	2 [1–3]	**0**.**005**	6 [3–10]	5 [2–10]	0.30
Number of recent different sexual partners**	1 [0–1]	1 [1–1]	**0**.**018**	1 [1–2]	1 [1–2]	0.69
Recent painful urination**	22 (51.1)	55 (35.3)	**0**.**058**	12 (42.9)	43 (42.6)	0.98
Recent genital discharge or ulcer**	15 (33.3)	25 (15.4)	**0**.**007**	4 (14.3)	13 (12.6)	0.81
Recent hospitalization**	6 (14.0)	19 (12.2)	0.76	1 (3.6)	5 (5.0)	0.76
Pregnant in the past three years**	20 (44.4)	79 (48.8)	0.61	---	---	---
Schistosome CAA infection	20 (44.4)	48 (29.6)	**0**.**061**	8 (28.6)	39 (37.9)	0.36

*Values shown are number (percent) or median [interquartile range] and are for available data. No variable was missing more than 3% of values.

**Time-dependent data were documented by the Kisesa observational HIV-1 cohort study at the time of the study participant’s HIV-1 diagnosis. Study participants were asked to report on behavior or events during the prior 12 months. For pregnancy data, participants reported the date of last delivery.

**Fig 2:**
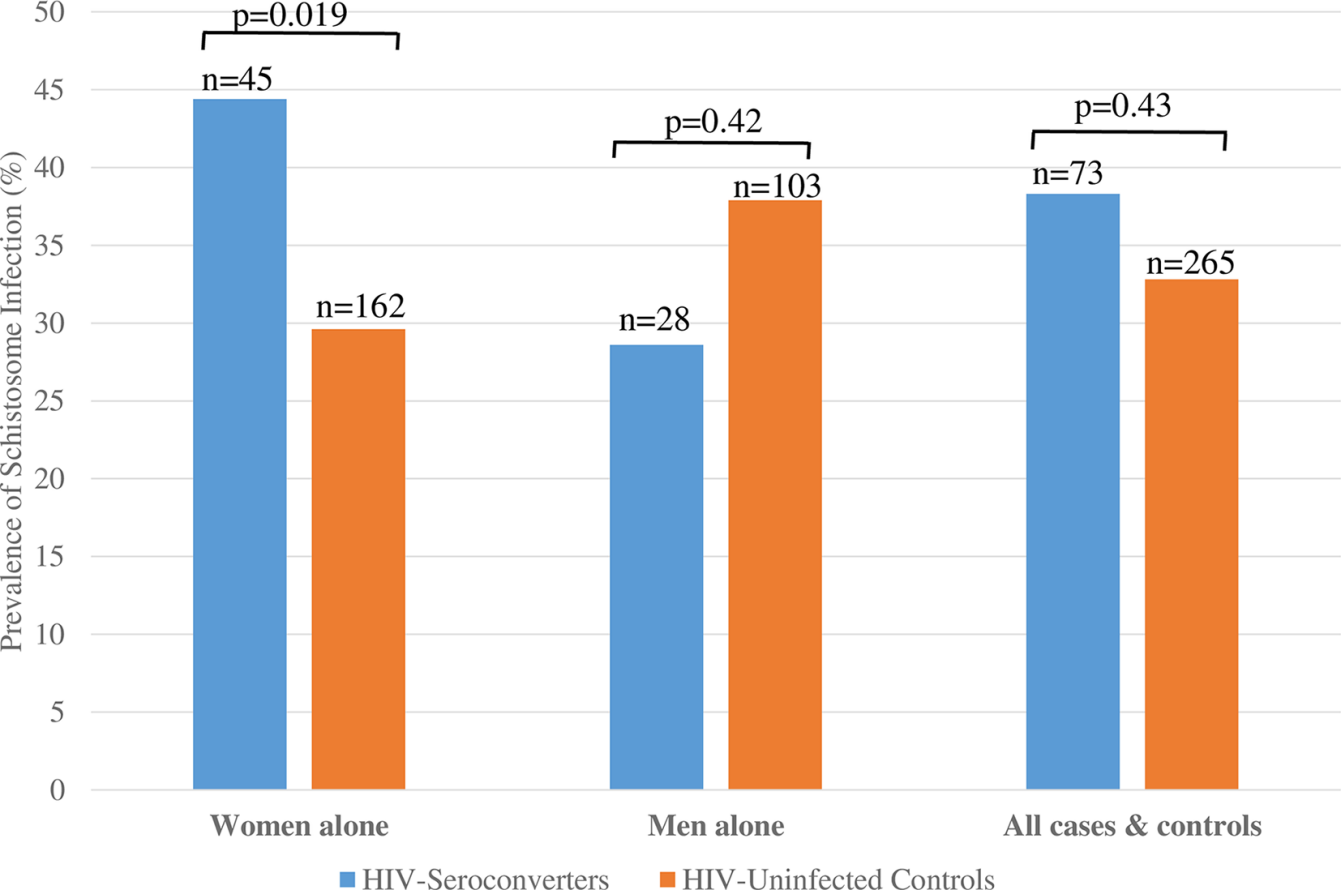
Prevalence of schistosome infection among HIV-1 seroconverters and matched controls, stratified by gender. In women, 44% of HIV-1 seroconverters had schistosome infection, compared to 30% of controls (p = 0.019). In men, 29% of HIV-1 seroconverters had schistosome infection, compared to 38% of controls (p = 0.42). These differences were not detectable when cases and controls were analyzed without stratification by gender.

In women, 20/45 HIV-1 seroconverters (44%) had schistosome infection at the time of HIV-1 acquisition, compared to 48/162 female HIV-uninfected controls (30%). After controlling for marital status and painful urination, women with schistosome infections had a 2.8-fold higher odds of incident HIV-1 infection than women without schistosome infections (OR = 2.8 [95% CI, 1.2–6.6], p = 0.019). In men, 8/28 HIV-1 seroconverters (29%) had schistosome infection at the time of HIV-1 acquisition, compared to 39/103 controls (38%), (OR = 0.7 [0.3–1.8], p = 0.42 after controlling for marital status). These data are shown in **Fig 2**.

### Study of whole blood viral load at HIV-1 seroconversion

For the study of HIV-1 RNA viral loads, we measured schistosome CAA in the dried blood spots of the 37 individuals who were diagnosed with HIV-1 infection in 2010. There were 22 women and 15 men. Fifteen of these (41%) had schistosome infection. There were no significant differences in baseline characteristics between those with and without schistosome infection. Study participants in both groups had had their last negative HIV test a median of 39 [interquartile range, 39–40] months before their first positive HIV test.

**Fig 3:**
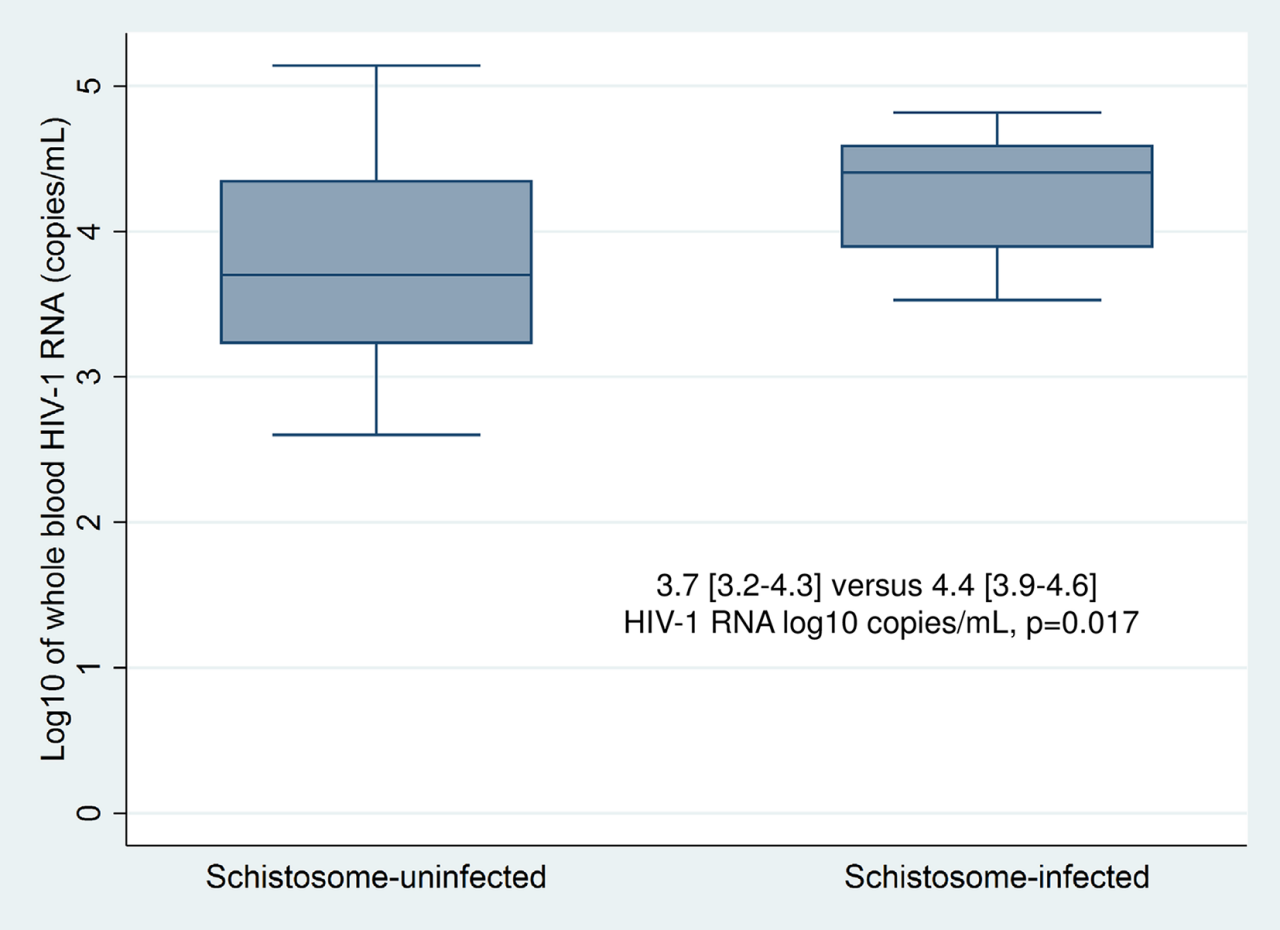
Median log_10_ whole blood HIV-1 RNA levels in recent HIV-1 seroconverters with and without schistosome infection. Summary plot depicting median and interquartile range of log_10_ of whole blood HIV-1 RNA level in copies/mL as quantitated from dried blood spots in recent HIV-1 seroconverters with or without schistosome infection. The median viral load was 4.4 [3.9–4.6] HIV-1 RNA log copies/mL in those with schistosome infection versus 3.7 [3.2–4.3] among those without (p = 0.017 by Wilcoxon rank-sum test).

We observed a significantly higher median whole blood HIV-1 RNA level in the 15 HIV-1 seroconverters with schistosome infection than in the 22 without schistosomiasis: 4.4 [3.9–4.6] log_10_ copies/mL versus 3.7 [3.2–4.3], p = 0.017 (**Fig 3**). On multivariable tobit regression by a backward selection procedure with an elimination criteria of p>0.05, the best-fit model included schistosome infection status and number of sexual partners in the past 12 months. This yielded an adjusted log_10_ HIV-1 RNA difference of 0.62 (β = 0.62 [0.23–1.01], p = 0.003).

The HIV-1 RNA level was 0.9 log_10_ copies/mL higher in the 11 women with schistosomiasis than the 11 women without (4.4 versus 3.5 log_10_ copies/mL, p = 0.02). The HIV-1 RNA level was 0.6 log_10_ copies/mL higher in the 4 men with schistosomiasis than the 11 men without (4.5 versus 3.9, p = 0.24).

Only one individual had an HIV-1 RNA level that was greater than 5 log_10_ copies/mL, and this person did not have schistosome infection. Two men had log_10_ HIV-1 RNA levels that were < 400 copies/mL and both of them were schistosome-uninfected; when these two values were removed from the analysis, the difference in median HIV-1 RNA levels remained significant (4.4 versus 3.9 log_10_ copies/mL in those with versus without schistosomiasis, p = 0.039). Of the 22 women included in the viral load analysis, two had been pregnant in the past year. Both women had delivered 5–6 months prior to dried blood spot collection, and the viral loads measured in their dried blood spots were both > 4 log_10_ copies/mL.

## Discussion

This study demonstrates that schistosome infection increases the susceptibility of women to HIV-1 acquisition and increases the HIV-1 viral load at HIV-1 seroconversion in those who become HIV-1-infected. Approximately 200 million schistosome-infected individuals live in African countries with generalized HIV-1 epidemics, and ~6 million of these are HIV-1 co-infected [1,34]. Our study suggests that interactions exist between HIV-1 and schistosomiasis that may play a critical role in HIV-1 transmission and disease progression in African countries.

Our study, conducted in an area endemic for *S*. *mansoni*, urges consideration of a causal relationship between *S*. *mansoni* infection and subsequent HIV-1 acquisition in women. Multiple studies already support the concept that parasite egg-induced damage to the urogenital mucosa may be a risk factor for HIV acquisition in *S*. *haematobium* infection. Autopsy studies suggest that *S*. *mansoni* eggs can also be found in the urogenital tract, particularly in heavily-infected individuals, and that eggs are not exclusively localized in the gastrointestinal tract as traditionally taught. In an autopsy series of individuals with mixed *S*. *haematobium/S*. *mansoni* infections in Egypt, 55% of total body *S*. *mansoni* eggs were in the intestinal mucosa and 24% were in the urogenital tract [35]. A case series in Tanzania showed that more than half of women with biopsy-confirmed *S*. *mansoni* infection of the cervix and no *S*. *haematobium* ova detected had visible lesions in the cervical tissue [8]. We therefore posit that *S*. *mansoni* has urogenital mucosal effects, in addition to its known intestinal mucosal effects, that increase women’s HIV-1 susceptibility. Plausible mechanisms include schistosome egg-induced physical breaches in genital mucosal tissue and recruitment of HIV-1-susceptible immune cells [2,5,36]. Anal sex could also contribute to our findings, as macaque studies support the hypothesis that *S*. *mansoni-*infected individuals likely have increased susceptibility to rectal HIV exposure [12].

We found that schistosomiasis increases the odds of HIV-1 acquisition in women but not in men. This strong gender effect may be due to differential effects of schistosome eggs in the genital mucosa of women versus men. In autopsy studies of the gastrointestinal and genitourinary organs in women with *S*. *haematobium* infection, the genital organs with the heaviest burdens of eggs were the cervix, vagina, and uterus [11,37,38]. These genital organs were also the most commonly affected organs in women with *S*. *mansoni* infection, with lower tissue egg burdens in *S*. *mansoni*-infected than in *S*. *haematobium-*infected women [35,37]. Multiple studies in men have demonstrated that the genital organs most affected by the eggs of both *S*. *haematobium* and *S*. *mansoni* are the seminal vesicles and prostate [11,35,39]—internal organs that are not exposed during sexual HIV-1 contact. This gender effect may explain why a prior study from Uganda reported no increased odds of HIV-1 seroconversion in *S*. *mansoni*-infected individuals [40]. The Uganda study had more men than women and did not examine differential effects by gender. Further, schistosome infections were three times more prevalent in men than in women (75% versus 21%). The study did find that history of anti-schistosome treatment was protective against incident HIV-1 infection.

We also found that schistosomiasis at the time of HIV-1 infection led to a 0.7 log_10_ increase in viral load at the time of HIV-1 seroconversion. A sustained 0.7 log_10_ HIV-1 viral load increase equates with an approximate doubling in infectivity among HIV-1-schistosome co-infected individuals and would be expected to accelerate time to symptomatic AIDS or death by 2–3 years [41]. This finding of increased HIV-1 RNA viral load at seroconversion is supported by studies from humans and mice showing that schistosome-induced immune alterations may impair the host’s ability to control viral replication. It is also supported by a recent Cochrane review suggesting that treatment of helminth infections in HIV-helminth co-infected individuals may decrease the HIV-1 RNA viral load [42]. Mouse models demonstrate that schistosome infections shift host immunity away from antiviral cytolytic T-helper (Th)-1 immune responses and towards a Th2-predominant state, thereby prompting reactivation of latent viruses [43,44].

Studies have suggested that HIV-1 viral load set-point may be affected by host mucosal inflammation [45,46]. In Ugandan HIV-1-serodiscordant couples, set-points were significantly higher in those who reported genital ulcer disease in the six months prior to HIV-1 infection than in those without genital disease [45]. South African women who had high levels of inflammatory genital cytokines before and 6 weeks after HIV-1 acquisition ultimately developed set-points that were significantly higher than those without genital inflammation [46]. Studies have shown that schistosomiasis also alters host mucosal immunity, and our study demonstrates that schistosomiasis increases viral loads at the time of HIV-1 seroconversion. An important future question, which we did not have power to answer, is whether the effect of schistosomiasis on viral load at HIV-1 seroconversion differs by gender, which would further implicate the genital mucosa as a critical mediator of the effect of schistosomiasis on HIV-1.

The single measurement of HIV-1 RNA level was likely to be reflective of the true viral load set-point for the large majority of the recent HIV-1 seroconverters in our study. Had additional samples been available, it would have been ideal to confirm values for HIV-1 viral load set-points in two separate samples collected between 6 weeks and 24 months after HIV seroconversion [15,25,26]. Acute HIV-1 seroconverters experience peak HIV-1 viremia a median of 13 days after HIV-1 RNA becomes detectable, and the HIV antibody test becomes positive a median of one day later [15]. These seroconverters would then be predicted to have a window lasting approximately 17 days during which the HIV antibody test would be positive and the HIV-1 RNA would not yet have reached a nadir or set-point [15]. Given the three-year HIV-testing intervals in our study, 1.6% of participants (less than 1 of the 37 HIV-seroconverters) would be predicted to have had a dried blood spot collected during this window. Indeed, only one person in our study had a viral load greater than 5 log_10_ copies/mL, and that person was schistosome-uninfected.

It is also unlikely that antiretroviral therapy use impacted our viral load analysis because we limited our analysis to individuals who HIV-seroconverted in 2010. In 2010, antiretroviral therapy was not yet widely available in the area and was only prescribed for individuals with CD4^+^ T-cell counts < 200 cells/μL or to HIV-infected mothers between 28 weeks of gestation and one week post-partum [23,24]. Only 17% of all HIV-infected pregnant mothers in Kisesa received this three-month course of antiretroviral therapy in 2010 [24]. The two HIV-infected women who delivered 5 and 6 months prior to dried blood spot collection had viral loads above 4 log_10_ copies/mL, suggesting that even if they did receive antiretroviral therapy around the time of delivery, they were not likely receiving antiretroviral therapy at the time of viral load measurement. Further, our sensitivity analysis showed significant differences in median viral loads even with removal of the two schistosome-uninfected individuals who had HIV-1 RNA levels less than 400 copies/mL and increases the robustness of our findings.

Because CAA testing cannot distinguish between schistosome species, we cannot determine with certainty whether *S*. *haematobium*, *S*. *mansoni*, or both increase susceptibility to HIV infection. It remains possible that our findings are driven by a small number of cases of *S*. *haematobium* infection. This seems unlikely given that multiple epidemiologic surveys have shown that the prevalence of *S*. *mansoni* is 20 times higher than *S*. *haematobium* in our population. Due to our utilization of archived samples, our study was limited by the small blood spot volume available and the lack of other samples for additional laboratory testing. We therefore relied on symptom report rather than laboratory confirmation of genital tract infections. A strength of our study is that ample demographic, behavioral, and symptom-report data allowed us to control at least partially for some of the other known HIV-1 risk factors. We were also unable to test for other helminth infections, including *Wuchereria bancrofti*, which has been shown to increase the risk of incident HIV infection in individuals in southern Tanzania [47]. In the district in northern Tanzania where we worked, it was recently determined that mass drug administration for elimination of *W*. *bancrofti* is not required due to low prevalence [48]. The definitive study to determine causality will be an interventional trial showing that treating schistosomiasis decreases incident HIV-1 infections and lowers viral load set-points.

In conclusion, we have demonstrated that chronic schistosome infection increases HIV-1 incidence in women and raises the HIV-1 viral load at the time of HIV-1-seroconversion. Praziquantel is an inexpensive, safe medication for schistosome infection [1]. Studies have shown that mass therapy can decrease the community prevalence of schistosome infection and that it may reverse urogenital tract pathology, particularly in younger individuals [49]. Robust prospective data to determine the effects of praziquantel treatment on tissue pathology in adolescents and adults is lacking. Our findings suggest that trials are warranted to determine the effectiveness of mass praziquantel treatment to decrease HIV-1 transmission and slow HIV-1 disease progression.

## Supporting information

S1 Checklist STROBE checklist. (PDF)

## References

[1] World Health Organization. Schistosomiasis Fact Sheet No 115 [Internet]. 2017 [cited 7 Feb 2017]. Available: http://www.who.int/topics/schistosomiasis/en/

[2] Mbabazi PS, Andan O, Fitzgerald DW, Chitsulo L, Engels D, Downs JA. Examining the relationship between urogenital schistosomiasis and HIV infection. PLoS Negl Trop Dis. 2011;5: e1396 doi: 10.1371/journal.pntd.0001396 22163056 10.1371/journal.pntd.0001396 PMC3232194

[3] Secor WE. Interactions between schistosomiasis and infection with HIV-1. Parasite Immunol. 2006;28: 597–603. doi: 10.1111/j.1365-3024.2006.00887.x 17042931 10.1111/j.1365-3024.2006.00887.x

[4] Downs JA, Mguta C, Kaatano GM, Mitchell KB, Bang H, Simplice H, et al Urogenital schistosomiasis in women of reproductive age in Tanzania’s Lake Victoria region. Am J Trop Med Hyg. 2011;84: 364–9. doi: 10.4269/ajtmh.2011.10-0585 21363971 10.4269/ajtmh.2011.10-0585 PMC3042809

[5] Kjetland EF, Ndhlovu PD, Gomo E, Mduluza T, Midzi N, Gwanzura L, et al Association between genital schistosomiasis and HIV in rural Zimbabwean women. AIDS. 2006;20: 593–600. doi: 10.1097/01.aids.0000210614.45212.0a 16470124 10.1097/01.aids.0000210614.45212.0a

[6] Downs JA, van Dam GJ, Changalucha JM, Corstjens PLAM, Peck RN, de Dood CJ, et al Association of schistosomiasis and HIV infection in Tanzania. Am J Trop Med Hyg. 2012;87: 868–73. doi: 10.4269/ajtmh.2012.12-0395 23033399 10.4269/ajtmh.2012.12-0395 PMC3516262

[7] Feldmeier H, Krantz I, Poggensee G. Female genital schistosomiasis as a risk-factor for the transmission of HIV. Int J STD AIDS. 1994;5: 368–72. Available: http://www.ncbi.nlm.nih.gov/pubmed/7819359 doi: 10.1177/095646249400500517 7819359 10.1177/095646249400500517

[8] Poggensee G, Krantz I, Kiwelu I, Diedrich T, Feldmeier H. Presence of Schistosoma mansoni eggs in the cervix uteri of women in Mwanga District, Tanzania. Trans R Soc Trop Med Hyg. 2001;95: 299–300. Available: http://www.ncbi.nlm.nih.gov/pubmed/11491002 11491002 10.1016/s0035-9203(01)90239-1

[9] Secor WE. The effects of schistosomiasis on HIV / AIDS infection, progression and transmission. Curr Opin HIV AIDS. 2012;7: 254–259. doi: 10.1097/COH.0b013e328351b9e3 22327410 10.1097/COH.0b013e328351b9e3 PMC11316515

[10] Stecher CW, Kallestrup P, Kjetland EF, Vennervald B, Petersen E. Considering treatment of male genital schistosomiasis as a tool for future HIV prevention: a systematic review. Int J Public Heal. 2015;60: 839–48. doi: 10.1007/s00038-015-0714-7 26298443 10.1007/s00038-015-0714-7

[11] Edington GM, Nwabuebo I, Junaid TA. The pathology of schistosomiasis in Ibadan, Nigeria with special reference to the appendix, brain, pancreas and genital organs. Trans R Soc Trop Med Hyg. 1975;69: 153–6. Available: http://www.ncbi.nlm.nih.gov/pubmed/1145708 1145708 10.1016/0035-9203(75)90027-9

[12] Chenine A-L, Shai-Kobiler E, Steele LN, Ong H, Augostini P, Song R, et al Acute Schistosoma mansoni infection increases susceptibility to systemic SHIV clade C infection in rhesus macaques after mucosal virus exposure. PLoS Negl Trop Dis. 2008;2: e265 doi: 10.1371/journal.pntd.0000265 18648516 10.1371/journal.pntd.0000265 PMC2447882

[13] Siddappa NB, Hemashettar G, Shanmuganathan V, Semenya AA, Sweeney ED, Paul KS, et al Schistosoma mansoni enhances host susceptibility to mucosal but not intravenous challenge by R5 Clade C SHIV. PLoS Negl Trop Dis. 2011;5: e1270 doi: 10.1371/journal.pntd.0001270 21829749 10.1371/journal.pntd.0001270 PMC3149020

[14] Chenine A-L, Buckley KA, Li P-L, Rasmussen RA, Ong H, Jiang S, et al Schistosoma mansoni infection promotes SHIV clade C replication in rhesus macaques. AIDS. 2005;19: 1793–7. Available: http://www.ncbi.nlm.nih.gov/pubmed/16227786 16227786 10.1097/01.aids.0000189857.51935.0b

[15] Robb ML, Eller LA, Kibuuka H, Rono K, Maganga L, Nitayaphan S, et al Prospective Study of Acute HIV-1 Infection in Adults in East Africa and Thailand. N Engl J Med. 2016;374: 2120–30. doi: 10.1056/NEJMoa1508952 27192360 10.1056/NEJMoa1508952 PMC5111628

[16] Mackelprang RD, Carrington M, Thomas KK, Hughes JP, Baeten JM, Wald A, et al Host genetic and viral determinants of HIV-1 RNA set point among HIV-1 seroconverters from sub-saharan Africa. J Virol. 2015;89: 2104–11. doi: 10.1128/JVI.01573-14 25473042 10.1128/JVI.01573-14 PMC4338863

[17] Mellors JW, Rinaldo CR, Gupta P, White RM, Todd JA, Kingsley LA. Prognosis in HIV-1 infection predicted by the quantity of virus in plasma. Science. 1996;272: 1167–70. Available: http://www.ncbi.nlm.nih.gov/pubmed/8638160 8638160 10.1126/science.272.5265.1167

[18] Quinn TC, Wawer MJ, Sewankambo N, Serwadda D, Li C, Wabwire-Mangen F, et al Viral load and heterosexual transmission of human immunodeficiency virus type 1. Rakai Project Study Group. N Engl J Med. 2000;342: 921–9. doi: 10.1056/NEJM200003303421303 10738050 10.1056/NEJM200003303421303

[19] Wambura M, Urassa M, Isingo R, Ndege M, Marston M, Slaymaker E, et al HIV prevalence and incidence in rural Tanzania: results from 10 years of follow-up in an open-cohort study. J Acquir Immune Defic Syndr. 2007;46: 616–23. doi: 10.1097/QAI.0b013e31815a571a 18043316 10.1097/QAI.0b013e31815a571a PMC2842883

[20] Mwaluko G, Urassa M, Isingo R, Zaba B, Boerma JT. Trends in HIV and sexual behaviour in a longitudinal study in a rural population in Tanzania, 1994–2000. AIDS. 2003;17: 2645–51. doi: 10.1097/01.aids.0000088225.55968.9d 14685059 10.1097/00002030-200312050-00012

[21] Downs JA, Corstjens PLAM, Mngara J, Lutonja P, Isingo R, Urassa M, et al Correlation of serum and dried blood spot results for quantitation of Schistosoma circulating anodic antigen: A proof of principle. Acta Trop. 2015;150: 59–63. doi: 10.1016/j.actatropica.2015.06.026 26149541 10.1016/j.actatropica.2015.06.026 PMC4592803

[22] Downs JA, de Dood CJ, Dee HE, McGeehan M, Khan H, Marenga A, et al Schistosomiasis and Human Immunodeficiency Virus in Men in Tanzania. Am J Trop Med Hyg. 2017;96: 856–62. doi: 10.4269/ajtmh.16-0897 28167600 10.4269/ajtmh.16-0897 PMC5392632

[23] Kanjala C, Michael D, Todd J, Slaymaker E, Calvert C, Isingo R, et al Using HIV-attributable mortality to assess the impact of antiretroviral therapy on adult mortality in rural Tanzania. Glob Heal Action. 2014;7: 21865 Available: http://www.pubmedcentral.nih.gov/articlerender.fcgi?artid=3962553&tool=pmcentrez&rendertype=abstract 10.3402/gha.v7.21865 PMC3962553 24656167

[24] Gourlay A, Wringe A, Todd J, Cawley C, Michael D, Machemba R, et al Uptake of services for prevention of mother-to-child transmission of HIV in a community cohort in rural Tanzania from 2005 to 2012. BMC Heal Serv Res. 2016;16: 4 doi: 10.1186/s12913-015-1249-6 26739028 10.1186/s12913-015-1249-6 PMC4702391

[25] Henrard DR, Phillips JF, Muenz LR, Blattner WA, Wiesner D, Eyster ME, et al Natural history of HIV-1 cell-free viremia. JAMA. 1995;274: 554–8. Available: http://www.ncbi.nlm.nih.gov/pubmed/7629984 7629984

[26] Farzadegan H, Henrard DR, Kleeberger CA, Schrager L, Kirby AJ, Saah AJ, et al Virologic and serologic markers of rapid progression to AIDS after HIV-1 seroconversion. J Acquir Immun Defic Syndr Hum Retrovirol. 1996;13: 448–55. Available: http://www.ncbi.nlm.nih.gov/pubmed/8970472 10.1097/00042560-199612150-00008 8970472

[27] de Water R, Fransen JA, Deelder AM. Ultrastructural localization of the circulating anodic antigen in the digestive tract of Schistosoma mansoni using monoclonal antibodies in an immunogold labeling procedure. Am J Trop Med Hyg. 1986;35: 549–58. Available: http://www.ncbi.nlm.nih.gov/pubmed/3518506 3518506 10.4269/ajtmh.1986.35.549

[28] van Dam GJ, Bogitsh BJ, van Zeyl RJ, Rotmans JP, Deelder AM. Schistosoma mansoni: in vitro and in vivo excretion of CAA and CCA by developing schistosomula and adult worms. J Parasitol. 1996;82: 557–64. Available: http://www.ncbi.nlm.nih.gov/pubmed/8691363 8691363

[29] Agnew A, Fulford AJ, De Jonge N, Krijger FW, Rodriguez-Chacon M, Gutsmann V, et al The relationship between worm burden and levels of a circulating antigen (CAA) of five species of Schistosoma in mice. Parasitology. 1995;111 (Pt 1): 67–76. Available: http://www.ncbi.nlm.nih.gov/pubmed/7609992 7609992 10.1017/s0031182000064611

[30] Polman K, Engels D, Fathers L, Deelder AM, Gryseels B. Day-to-day fluctuation of schistosome circulating antigen levels in serum and urine of humans infected with Schistosoma mansoni in Burundi. Am J Trop Med Hyg. 1998;59: 150–4. Available: http://www.ncbi.nlm.nih.gov/pubmed/9684644 9684644 10.4269/ajtmh.1998.59.150

[31] Monleau M, Montavon C, Laurent C, Segondy M, Montes B, Delaporte E, et al Evaluation of different RNA extraction methods and storage conditions of dried plasma or blood spots for Human Immunodeficiency Virus type 1 RNA quantification and PCR amplification for drug resistance testing. J Clin Microbiol. 2009;47: 1107–1118. doi: 10.1128/JCM.02255-08 19193835 10.1128/JCM.02255-08 PMC2668360

[32] Arredondo M, Garrido C, Parkin N, Zahonero N, Bertagnolio S, Soriano V, et al Comparison of HIV-1 RNA measurements obtained by using plasma and dried blood spots in the automated abbott real-time viral load assay. J Clin Microbiol. 2012;50: 569–72. doi: 10.1128/JCM.00418-11 22170904 10.1128/JCM.00418-11 PMC3295109

[33] Rutstein SE, Kamwendo D, Lugali L, Thengolose I, Tegha G, Fiscus SA, et al Measures of viral load using Abbott RealTime HIV-1 Assay on venous and fingerstick dried blood spots from provider-collected specimens in Malawian District Hospitals. J Clin Virol. 2014;60: 392–8. doi: 10.1016/j.jcv.2014.05.005 24906641 10.1016/j.jcv.2014.05.005 PMC4073118

[34] Ndeffo Mbah ML, Poolman EM, Drain PK, Coffee MP, van der Werf MJ, Galvani AP. HIV and Schistosoma haematobium prevalences correlate in sub-Saharan Africa. Trop Med Int Heal. 2013;18: 1174–9. doi: 10.1111/tmi.12165 23952297 10.1111/tmi.12165 PMC4797643

[35] Cheever AW, Kamel IA, Elwi AM, Mosimann JE, Danner R. Schistosoma mansoni and S. haematobium infections in Egypt. II. Quantitative parasitological findings at necropsy. Am J Trop Med Hyg. 1977;26: 702–16. Available: http://www.ncbi.nlm.nih.gov/pubmed/889013 889013 10.4269/ajtmh.1977.26.702

[36] Jourdan PM, Holmen SD, Gundersen SG, Roald B, Kjetland EF. HIV target cells in Schistosoma haematobium-infected female genital mucosa. Am J Trop Med Hyg. 2011;85: 1060–4. doi: 10.4269/ajtmh.2011.11-0135 22144444 10.4269/ajtmh.2011.11-0135 PMC3225152

[37] Gelfand M, Ross WF. II. The distribution of schistosome ova in the genito-urinary tract in subjects of bilharziasis. Trans R Soc Trop Med Hyg. 1953;47: 218–20. Available: http://www.ncbi.nlm.nih.gov/pubmed/13077721 13077721 10.1016/0035-9203(53)90006-6

[38] Gelfand M, Ross MD, Blair DM, Weber MC. Distribution and extent of schistosomiasis in female pelvic organs, with special reference to the genital tract, as determined at autopsy. Am J Trop Med Hyg. 1971;20: 846–9. Available: http://www.ncbi.nlm.nih.gov/pubmed/5131693 5131693 10.4269/ajtmh.1971.20.846

[39] Smith JH, Kamel IA, Elwi A, Von Lichtenberg F. A quantitative post mortem analysis of urinary schistosomiasis in Egypt. I. Pathology and pathogenesis. Am J Trop Med Hyg. 1974;23: 1054–71. Available: http://www.ncbi.nlm.nih.gov/pubmed/4429178 4429178 10.4269/ajtmh.1974.23.1054

[40] Ssetaala A, Nakiyingi-Miiro J, Asiki G, Kyakuwa N, Mpendo J, Van Dam GJ, et al Schistosoma mansoni and HIV acquisition in fishing communities of Lake Victoria, Uganda: a nested case-control study. Trop Med Int Heal. 2015;20: 1190–1195. doi: 10.1111/tmi.12531 25940951 10.1111/tmi.12531 PMC4529482

[41] Modjarrad K, Chamot E, Vermund SH. Impact of small reductions in plasma HIV RNA levels on the risk of heterosexual transmission and disease progression. AIDS. 2008;22: 2179–85. doi: 10.1097/QAD.0b013e328312c756 18832881 10.1097/QAD.0b013e328312c756 PMC2661869

[42] Means AR, Burns P, Sinclair D, Walson JL. Antihelminthics in helminth-endemic areas: effects on HIV disease progression. Means AR, editor. Cochrane Database Syst Rev. Chichester, UK: John Wiley & Sons, Ltd; 2016;4: CD006419 doi: 10.1002/14651858.CD006419.pub4 27075622 10.1002/14651858.CD006419.pub4 PMC4963621

[43] Osborne LC, Monticelli LA, Nice TJ, Sutherland TE, Siracusa MC, Hepworth MR, et al Virus-helminth coinfection reveals a microbiota-independent mechanism of immunomodulation. Science. 2014;345: 578–82. doi: 10.1126/science.1256942 25082704 10.1126/science.1256942 PMC4548887

[44] Reese TA, Wakeman BS, Choi HS, Hufford MM, Huang SC, Zhang X, et al Coinfection. Helminth infection reactivates latent γ-herpesvirus via cytokine competition at a viral promoter. Science. 2014;345: 573–7. doi: 10.1126/science.1254517 24968940 10.1126/science.1254517 PMC4531374

[45] Hollingsworth TD, Laeyendecker O, Shirreff G, Donnelly CA, Serwadda D, Wawer MJ, et al HIV-1 transmitting couples have similar viral load set-points in Rakai, Uganda. PLoS Pathog. 2010;6: e1000876 doi: 10.1371/journal.ppat.1000876 20463808 10.1371/journal.ppat.1000876 PMC2865511

[46] Roberts L, Passmore J-AS, Mlisana K, Williamson C, Little F, Bebell LM, et al Genital tract inflammation during early HIV-1 infection predicts higher plasma viral load set point in women. J Infect Dis. 2012;205: 194–203. doi: 10.1093/infdis/jir715 22190580 10.1093/infdis/jir715 PMC3244362

[47] Kroidl I, Saathof E, Maganga L, Makunde W, Hoerauf A, Geldmacher C, et al Effect of Wuchereria bancrofti infection on HIV incidence in southwest Tanzania (EMINI): a prospective cohort study. Lancet. 2016;388: 1912–20. doi: 10.1016/S0140-6736(16)31252-1 27495354 10.1016/S0140-6736(16)31252-1

[48] Chikawe M, Rebollo M, Nshala A, Uisso C, Kazyoba P, Crowley K, et al. Steps towards elimination: Re-evaluation of lymphatic filariasis prevalence in Tanzania [Internet]. Dar es Salaam; 2016 [cited 7 May 2017]. Available: https://imaworldhealth.org/wp-content/uploads/2016/11/Steps-Towards-Elimination-11.11.16.pdf

[49] Kjetland EF, Ndhlovu PD, Kurewa EN, Midzi N, Gomo E, Mduluza T, et al Prevention of gynecologic contact bleeding and genital sandy patches by childhood anti-schistosomal treatment. Am J Trop Med Hyg. 2008;79: 79–83. Available: http://www.ncbi.nlm.nih.gov/pubmed/18606767 18606767

